# Potential applications of ficin in the production of traditional cheeses and protein hydrolysates

**DOI:** 10.3168/jdsc.2020-0073

**Published:** 2021-06-03

**Authors:** Mohammed Aider

**Affiliations:** 1 Department of Soil Sciences and Agri-Food Engineering, Université Laval, Québec, QC, G1V 0A6, Canada; 2 Institute of Nutrition and Functional Foods (INAF), Université Laval, Québec, QC, G1V 0A6, Canada

## Abstract

Using proteolytic enzymes extracted from plant materials is a promising way to ensure the sustainability of the food industry. This is particularly true for the dairy industry, especially in cheesemaking and the production of different milk protein hydrolysates for special food applications, particularly nutrition for infants, older adults, and people with food allergies. Ficin, a cysteyl protease isolated from the latex of the fig tree (*Ficus carica*), is characterized by strong enzymatic activity and can be used for milk clotting and protein hydrolysis for application in different foods. In particular, it can be used for milk protein hydrolysis to produce ingredients with reduced or eliminated allergenicity and improved bioavailability. Ficin can also be used as an active and effective replacement for calf rennet in cheesemaking, such as in traditional Cacioricotta and Teleme cheeses. It can also be used to produce protein hydrolysates with low or no allergenicity for application in infant formula and geriatric nutrition. This work provides an overview of ficin, a plant-derived protease, with an emphasis on its potential application in the production of some traditional cheeses and milk protein hydrolysates for special food applications.

## Highlights

• Ficin is a proteolytic enzyme (a cysteine protease) contained in fig tree (*Ficus carica*) latex.
• Ficin is effective for protein hydrolysis and as a clotting agent for cheesemaking.
• Ficin can be used to produce milk protein hydrolysates with potentially reduced allergenicity.
• Ficin can be used to produce milk protein ingredients for infant and geriatric nutrition.

## Graphical Abstract

**Figure:**
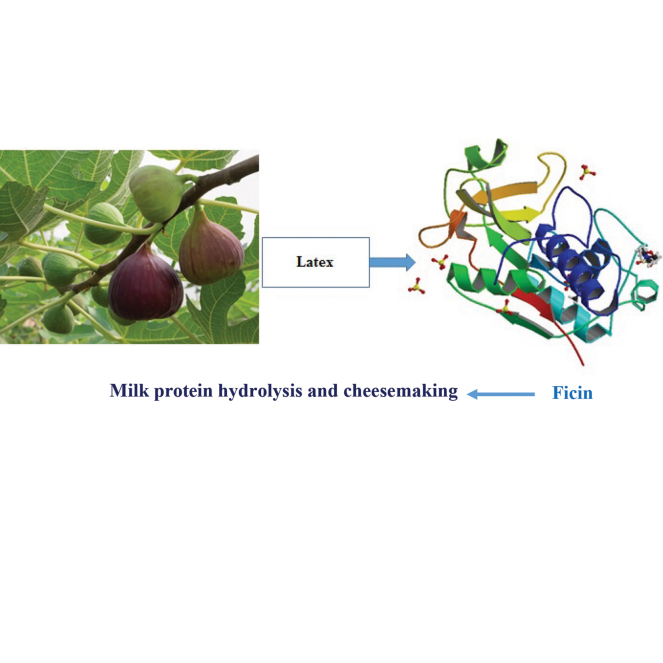
**Summary:** Ficin is a cysteine protease that is extracted from the latex of the fig tree (*Ficus carica*). It has strong catalytic activity that is pH dependent and it can be used in the dairy industry to produce milk protein hydrolysates for different applications, such as ingredients in infant formula and in geriatric nutrition formulas. These specific applications are very important because hydrolysis of milk proteins can significantly reduce the risk of allergenicity. Moreover, ficin can be successfully used in cheese making as clotting agent. This is a particularly interesting option for traditional cheeses and for cheeses made from milk with a high protein content such as ewe milk.

To overcome ethical issues in regards to calves and bioengineered chymosin, plant-derived proteases can be used to coagulate milk. One of the most promising plant-derived proteases is ficin, which is found in the latex of figs (*Ficus carica*). It belongs to the group of cysteine (Cys) endopeptidases that includes papain, bromelain, calpain, cathepsin B, and chymopapain (Singleton and Buttle, 2013; Gagaoua et al., 2014). Ficin can also be used to produce protein hydrolysates and in different food applications, such as improving tenderness in meat products. Ficin has also been used as a chill-proofing agent for beer, a dough conditioner, a rennet substitute, and a processing aid for precooked cereals (Arshad et al., 2016).

This work provides an overview of ficin with an emphasis on its potential as an effective protease in cheesemaking and in the production of milk protein hydrolysates with reduced allergenicity for special food applications, such as infant formula and geriatric nutrition. We focus on geriatric nutrition because as people age, metabolism activity is significantly reduced and the assimilation of highly complex molecules becomes difficult. In this context, the use of protein hydrolysates could help improve the bioavailability of nitrogen-containing nutrients (Vijaykrishnaraj and Prabhasankar, 2015).

Ficin is a cysteyl protease isolated from the latex of the fig tree (Haesaerts et al., 2015). A green fig weighing 10 to 15 g contains 100 to 150 mg of proteases. The optimal pH and temperature of ficin's proteolytic activity are 5.0 to 8.0 and 45 to 55°C, respectively (Wahyuni et al., 2017; Yang et al., 2017). Currently, only 3 fragments of ficin have been studied: a fragment around the Cys catalytic site, a catalytic fragment around His, and the N-terminal fragment. The AA sequence determined for the active site residues is almost identical to the sequence found in papain (Milošević et al., 2019; Figure 1).

**Figure 1:**
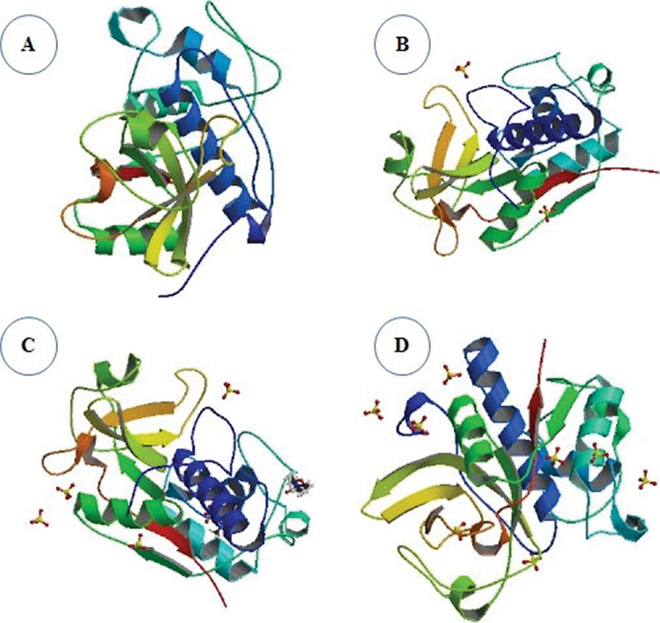
Molecular structure of ficin in its A, B, C, and D isoforms. Ficin isoform A has a total structure molecular weight of 47.20 kDa; isoform B has a total weight of 48.56 kDa; isoform C has a total weight of 25.12 kDa; and isoform D has a total weight of 24.50 kDa. Illustrations are from Berman et al. (2000) and are reproduced under a Creative Commons CC BY license.

Ficin can be used for milk coagulation and protein hydrolysis (Siar et al., 2020) and in immunohematology for the identification for irregular antibodies (Takeshita et al., 2010; Santis et al., 2019). Several studies have been carried out on the purification and biochemical characterization of ficin; however, few studies are available on the structural properties of ficin compared with papain and other related proteases. Ficin is a unique polypeptide chain with a molecular weight of 23.1 kDa. It belongs to the family of cysteine proteases (Reddy and Lerner, 2010) and is made of 210 AA residues. Its active site consists of 2 AA: Cys (Cys-25) and His (His-159). The enzyme is active at neutral pH, and its complete inactivation occurs below pH 3.0 (Milošević et al., 2019). Ficin activity is highest at pH 7, and its complete inactivation at pH 3 implies that it is safe for the human gastrointestinal tract. Moreover, it is metabolized with other proteins in food after being hydrolyzed. The pH-induced denaturation of ficin leads to a partially folded structure. The unfolded part of ficin at low pH shows characteristics of a molten globule corresponding to an intermediate state. Ficin requires cysteine or other reducing agents for activation, but it is inhibited by chicken cysteine. Moreover, among similar plant proteases, ficin has the lowest temperature of inactivation (~70°C; Devaraj et al., 2008). Ficin belongs to the group of cysteyl proteases, the catalytic mechanism of which involves a Cys group in the active site. Cysteine proteases, also known as thiol proteases, catalyze the breakdown of proteins by cleaving peptide bonds using a nucleophilic thiol from a cysteine of a protein. Proteases from *Ficus carica* have a wide range of specificity toward basic and neutral AA such as Gly, Val, Leu, Ala, Ser, Asn, Arg, and His. Ficin cleaves proteins at Tyr, Phe, and Val bonds.

Ficins from the latex of different fig trees can be purified using the following procedure. The first step consists of removing the gum from the aqueous solution by centrifugation. This is followed by dialyzing and concentrating the aqueous phase, which can then be eluted through an ion exchange resin. The enzymatic system of the latex is composed of 4 to 10 ficin fractions (Zare et al., 2013), and its relative activity has been demonstrated by using 2 inhibitors: iodoacetamide and potassium tetrathionate. Iodoacetamide and potassium tetrathionate inhibited enzyme activity by more than 90%, confirming that ficins are cysteine proteases (Azarkan et al., 2011).

In the Kabylia region of Algeria, ficin is extracted from fig tree leaves and used fresh to prepare *agugli*, a fresh soft cheese made with ewe milk (Gagaoua and Hafid, 2016). Bornaz et al. (2010) studied the coagulation of ewe milk using plant-sourced coagulants such as artichoke extract (*Cynara scolymus* L. ‘Blanca') and ficin as crude latex from the fig tree (*Ficus carica* L.) without any treatment. A turbidimetric method was used to evaluate and compare the coagulation properties of these enzymes with those of chymosin. The results showed that both the cynara coagulant and chymosin produced a sigmoidal increase in turbidity of the milk with 3 distinct phases, and the coagulation kinetics were substantially affected by all of the coagulants. Plant coagulants induced a shorter gelation time than chymosin but required more time for restructuring (at the end of coagulation). The coagulum obtained with ficin had a higher yield due to its high water-retention capacity. The authors reported that the overall sensory attributes were not significantly different among coagulants, but differences in color were observed between the cheeses. Color was assessed by panelists by means of a hedonic scale ranging from 1 to 10. The hedonic evaluation showed that ficin curds were lighter than those obtained using cynara extract, with mean values of 4 and 3, respectively. Color scores of ficin curds showed less variation among panelists than the 2 other cheeses, and no significant differences were observed among the 3 types of curds in regard to odor, taste, aftertaste, bitterness, texture, or overall acceptance (Bornaz et al., 2010). Low et al. (2006) used regular and UF (1×, 2×, and 4× concentrated) skim milk that was clotted by calf rennet, ficin, and papain. The clotting properties, curd casein profiles, and free AA contents were determined, and their results showed that UF milks coagulated faster and formed firmer curds irrespective of protein concentration. Furthermore, both ficin and papain had a more significant effect on proteolysis in curds formed from regular and 1× UF milk than in those formed from 2× or 4× UF milk. They reported that the UF process may cause structural changes in proteins or other milk constituents, with a resultant change in clotting properties and proteolysis of the casein. Moreover, they found that ficin had excessive proteolytic activity, leading to losses of peptides in the whey and thus lowering cheese yield (Low et al., 2006). Some of the difference between these 2 studies could be attributed to the milks used. The first study (Bornaz et al., 2010) used ewe milk, whereas the second study (Low et al., 2006) used regular and UF bovine skim milk. Ewe milk contains much more fat (7–7.5% vs. 3.5–4.5%) and total proteins (3–3.5% vs. 5.5–6.5%) and more casein than bovine milk (Antonič et al., 2013; Harthan and Cherney, 2017). Thus, the use of ficin for milk clotting in cheesemaking might be more suitable with for products made from ewe milk.

The use of whole fig latex as a source of ficin must be studied in more detail because its effectiveness for milk clotting depends on purity of the ficin. Raskovic et al. (2016) reported that comparison of latex samples in different time periods showed a uniform increase in protein concentration as time increased. The content of collagenolytic protease did not differ significantly in the latex samples, whereas ficin content decreased. They also reported that ficin-specific activity toward casein was highest (~80 U/mg) at the beginning of fruit development. The results they obtained with milk coagulation showed that specific milk-clotting activity increased, as did the abundance of casein bands in the clots. Specific chitinolytic activity at the beginning of flowering was 6.5 times higher than that when the fruits were ripe. They also found that whole-latex antifungal activity is highest in spring (Raskovic et al., 2016). Based on the findings of that study, it seems necessary to separate ficin from other enzymes contained in the latex to enhance its effectiveness for milk clotting and to collect the latex when the ficin content is highest.

The purity of ficin is an important factor in its suitability for cheesemaking. In a study by Akar and Fadiloglu (1999), fig tree latex (containing ficin and other proteases) was stepwise purified using ion exchange coupled with gel filtration chromatography and used in the production of Teleme cheese. The ratio of ficin milk clotting to its proteolytic activity increased from 1.97 to 3.1 following ion exchange chromatography and to 7.4 following gel filtration. Moreover, the Teleme cheese made with purified ficin had better chemical and sensory properties than that made with crude fig latex. The protein content of Teleme made with the fig latex and the purified ficin fraction was 3.90 and 6.50%, respectively. Furthermore, syneresis in Teleme decreased from 95% to 85% upon purification of the proteolytic enzymes. The authors stated that reducing proteolytic activity appears to be essential for improving the quality of Teleme produced using ficin for milk clotting (Akar and Fadiloglu, 1999).

Faccia et al. (2012) compared the clotting of goat milk using ficin from latex caprifig branches and calf rennet in the production of Cacioricotta, a traditional Italian goat cheese, which is produced using overheated milk at 90°C and without bacterial starters. The study was based on the quantification of the water-soluble, 15% trichloroacetic acid-soluble, and free AA fractions, reversed-phase (**RP**)-HPLC separation of low-molecular-weight peptides, and their identification by MALDI-TOF MS. The use of fig latex was associated with higher amounts of nitrogen-containing fractions and with RP-HPLC chromatograms that were highly rich in peptides. The use of calf rennet was associated with an almost complete lack of peptides in RP-HPLC chromatograms. This study confirmed the strong proteolytic activity of the enzymes contained in fig tree latex despite the intense overheating of the milk, which is considered to cause some reduction in the rate of casein hydrolysis during goat milk clotting (Faccia et al., 2012). In my opinion, the results of this study indicate that goat milk can be effectively used in developing dairy products with significantly reduced allergenicity and with milk proteins that are more easily assimilated by the human gastrointestinal tract. These features are particularly interesting in infant feeding. Cow milk is not suitable for infant feeding because of the difficulty of digesting casein micelles, which have large particle size for the digestive systems of infants less than 1 yr of age, and the associated problems with high casein and calcium intake. In a study by Ziegler (2007), feeding cow milk had adverse effects on iron nutrition in infants and young children, and several mechanisms were identified that may act synergistically. One of the identified factors is that cow milk provides high amounts of calcium and casein, both of which inhibit the absorption of dietary non-heme iron. Infants fed cow milk receive much more protein and minerals than they need, and the excess has to be excreted in urine. The high renal solute load leads to higher urine concentration during the feeding of cow milk than during the feeding of breast milk or formula. When fluid intakes are low or when extra-renal water losses are high, the renal concentrating ability of infants may be insufficient to maintain water balance in the face of high water use for excretion of the high-renal-load solute. The resulting negative water balance, if prolonged, can lead to serious dehydration (Ziegler, 2007). According to Ziegler (2007), deep hydrolysis of casein micelles by ficin would enhance their digestibility and reduce the amount of calcium fixed to the micelles.

Enzymatic hydrolysis of milk proteins is aimed at obtaining products with low allergenicity and high nutritional value and bioavailability (Xie et al., 2013). The positive physiological effect of consuming hydrolyzed proteins is caused by the better absorption of short-chain peptides in the gastrointestinal tract compared with absorption of native or high-molecular-weight proteins. Obtaining biologically active peptides, particularly those with antihypertensive, antimicrobial, immunomodulation, antifungal, and other active effects, is of particular interest for the food and pharmaceutical industries (Pihlanto-Leppälä et al., 2000). Milk protein hydrolysates are in demand as ingredients in infant formula and preventive and therapeutic baby foods.

The main characteristics of enzymatic hydrolysates are the degree of protein hydrolysis, the peptide composition, and the residual antigenicity, which is the amount of undigested protein that retains the ability to interact with antibodies. According to the degree of hydrolysis, 2 types of hydrolysates can be distinguished: partial hydrolysates containing peptides of various molecular weights and a minimum amount of free AA, and highly hydrolyzed fractions characterized by short-chain peptides and free AA. The residual antigenicity of partially hydrolyzed proteins used in prophylactic food mixtures must be at least 10^−3^ relative units, whereas that of highly hydrolyzed proteins, which are usually used in therapeutic food products, is 10^−6^ to 10^−4^ relative units, indicating that they are 10^4^ to 10^6^ times less antigenic than native proteins. Moreover, in severe cases of food allergies, only AA without antigenic properties are used. However, the significant drawback of highly hydrolyzed protein and mixtures of free AA is their pronounced bitter taste. Such ingredients are effectively used as a nitrogen source in microbiological culture media. Also, it has been reported that the use of partial protein hydrolysates with acceptable organoleptic characteristics is suitable in clinical geriatric nutrition. The difficulty of obtaining milk protein hydrolysates is associated with the choice of highly active enzymes for the efficient breakdown of milk proteins—in particular, β-LG, α-LA, and BSA, which are characterized by a compact globular structure that affects their relative resistance to proteolysis. Increases in the degree of hydrolysis of whey proteins and the yield of the peptide fraction and a decrease in their antigenic properties are achieved by establishing optimal conditions such as heat treatment before enzymatic hydrolysis. Allergenic proteins can be decreased or eliminated using a wide range of proteases. Ficin, the activity of which is very similar to that of papain, can be effectively used to obtain highly hydrolyzed proteins with reduced allergenicity (Fadýloğlu, 2001). Protein hydrolysis using ficin, which has strong proteolytic activity, can reduce the probability of protein allergenicity by affecting the integrity of epitopes recognized by IgG or IgE antibodies. These modifications have potential importance in allergenicity reduction because they may affect the ability of antibodies to bind to the modified protein. In the case of IgE antibody binding, this may result in an altered capacity to induce an allergic reaction. In the case of IgE-mediated milk protein allergy, the effect of ficin in reducing allergenicity could be attributed to its potential effect on the capacity of a modified or hydrolyzed protein to stimulate the production of IgE antibodies (Verhoeckx et al., 2015). However, more detailed studies of the specific features of hydrolysis of milk proteins by ficin are necessary to establish the optimal parameters for obtaining hydrolysates with a given peptide composition and immunochemical properties to be used as ingredients in functional foods. According to studies carried out on the hydrolysis of whey proteins with different proteases, such as alkalase, trypsin, pepsin, and ficin, different profiles of hydrolysates can be obtained. For the studied proteases, different substrate specificity was shown for β-LG, α-LA, and BSA. β-Lactoglobulin is resistant to pepsin hydrolysis under acidic condition with pH 2, which is in the range of stomach pH, and ficin effectively hydrolyzes this protein in neutral and alkaline conditions.

In light of the information published on ficin and its potential food applications, 2 main possibilities stand out. First, the protein composition of ficin makes it suitable for use as a proteolytic enzyme in the production of certain types of cheese, in particular those made with sheep milk. Ficin could also be used in the manufacture of cheese based on UF cow milk. Second, ficin could be successfully used for the production of milk protein hydrolysates to obtain ingredients with reduced allergenicity and better bioavailability for applications in infant formulas and in geriatric nutrition.
